# The effect of network mixing patterns on epidemic dynamics and the efficacy of disease contact tracing

**DOI:** 10.1098/rsif.2007.1272

**Published:** 2007-12-04

**Authors:** Istvan Z Kiss, Darren M Green, Rowland R Kao

**Affiliations:** 1 Department of Mathematics, University of SussexFalmer, Brighton BN1 9RF, UK; 2 Institute of Aquaculture, University of StirlingStirling, Stirlingshire FK9 4LA, UK; 3 Faculty of Veterinary Medicine, Institute of Comparative Medicine, University of GlasgowGlasgow G61 1QH, UK

**Keywords:** contact tracing, mixing pattern, networks, epidemics

## Abstract

In networks, nodes may preferentially contact other nodes with similar (assortatively mixed) or dissimilar (disassortatively mixed) numbers of contacts. Different patterns of contact support different epidemic dynamics, potentially affecting the efficacy of control measures such as contact tracing, which aims to identify and isolate nodes with infectious contacts. We used stochastic simulations to investigate the effects of mixing patterns on epidemic dynamics and contact-tracing efficacy. For uncontrolled epidemics, outbreaks occur at lower infection rates for more assortatively mixed networks, with faster initial epidemic growth rate and shorter epidemic duration than for disassortatively mixed networks. Contact tracing performs better for assortative mixing where epidemic size is large and tracing rate low, but it performs better for disassortative mixing at higher contact rates. For assortatively mixed networks, disease spreads first to highly connected nodes, but this is balanced by contact tracing quickly identifying these same nodes. The converse is true for disassortative mixing, where both disease and tracing are less likely to target highly connected nodes. For small epidemics, contact tracing is more effective on disassortative networks due to the greater resilience of assortative networks to link removal. Multi-step contact tracing is more effective than single-step tracing for assortative mixing, but this effect is smaller for disassortatively mixed networks.

## 1. Introduction

For a wide range of epidemic and epizootic diseases, individuals can be usefully modelled as nodes in a network, where the network links represent potentially infectious contacts between individuals. This network representation applies to many complex systems such as the Internet, the World Wide Web (Albert & Barabási 2002), social and transportation networks (Liljeros *et al.* 2001; Jones & Handcock 2003; Hufnagel *et al.* 2004) and livestock movement networks (Kiss *et al.* 2006*b*; Robinson *et al.* 2007). Models that use explicit contact structures between individuals, households, cities, regions, countries or farms have been used to analyse the spread of human diseases, such as SARS (Hufnagel *et al.* 2004; Meyers *et al.* 2005) and pandemic influenza (Eubank *et al.* 2004; Ferguson *et al.* 2005), and animal diseases, such as foot-and-mouth disease (FMD; Green *et al.* 2006*a*; Kao *et al.* 2006; Kiss *et al.* 2006*a*) and avian influenza (Le Menach *et al.* 2006). Much attention is now focused on analysing network properties and the dynamic processes they support (for reviews, see Newman (2003*b*) and Keeling & Eames (2005)), as well as how to effectively control these processes.

Contact tracing is commonly used to identify individuals that have been in contact with infectious individuals, to reduce the number of potential onward infections from traced individuals. Contact tracing was successful in the 2003 SARS epidemic (Lipsitch *et al.* 2003), but unsuccessful in the 2001 FMD epidemic in the UK (Ferguson *et al.* 2001; Keeling *et al.* 2001; Kao 2003). For simple unstructured populations, contact tracing has been modelled using a combination of branching process analysis complemented by stochastic simulation models (Müller *et al.* 2000). However, in more highly structured contact networks, the properties of the network markedly influence the efficacy of contact tracing. For example, contact tracing is more effective on clustered networks than on random networks (Eames & Keeling 2003; Kiss *et al.* 2005), and on scale-free networks a higher tracing effort is needed to control an epidemic than on random Poisson networks (Kiss *et al.* 2006*b*).

In this paper, we examine the impact of departure from the common assumption of proportionate (random) mixing upon the efficacy of contact tracing. Proportionate mixing assumes that the probability of any two nodes being connected is proportional to the product of their number of contacts. This is unlikely in many cases where ‘like attaches to like, assortatively mixed’ (e.g. STI transmission; Anderson *et al.* 1990; Catania *et al.* 1992) or where ‘opposites attract, disassortatively mixed’ (e.g. livestock trading among farms and markets in the UK (Kiss *et al.* 2006*a*); correlation properties of the Internet (Pastor-Satorras *et al.* 2001)). The empirical evidence and importance of connectivity correlations have led to numerous network- and differential equation-based models (Boguñá *et al.* 2003*a*; Newman 2003*a*; Barthélemy *et al.* 2005). Many of these models focus on the effect of connectivity correlations on the epidemic threshold, initial growth rate and hierarchical spread. For example, it has been shown that epidemics on networks characterized by high node degree variance grow rapidly, and in the limiting case of infinite variance, instantaneously, independently of the mixing pattern (Boguñá *et al.* 2003*b*). The initial growth of the epidemic changes from exponential to power law if non-random mixing is combined with small-world properties, with the power-law exponent determined by the average node distance on the network (Vazquez 2006). While in the limit of infinite populations, the implications of preferential mixing for disease invasion are well known, here we focus on disease control and consider the efficacy of contact tracing for different mixing patterns. For networks that are poorly characterized, the efficacy of contact tracing is difficult to determine without exact knowledge of the underlying contact network, i.e. who is connected to whom? By analysing disease transmission on theoretical networks with different mixing patterns, we aim to identify the implications of non-random mixing for epidemic dynamics and control strategies.

## 2. Methods

### 2.1 Disease transmission

The network-based disease transmission and contact-tracing model is based on models considered by Huerta & Tsimring (2002), Eames & Keeling (2003) and Kiss *et al.* (2005, 2006*a*). Each node in the network is classified according to one of four states of disease progression: susceptible nodes (*S*); nodes at once infected and infectious (*I*); nodes ‘triggering’ contact tracing (*T*), which are identified as being infectious, are immediately isolated and initiate tracing; and, finally, removed nodes (*R*), which are no longer infectious and do not initiate tracing. The transitions between states are depicted in figure 1.

*Infection S→I*. The epidemic is seeded with one or more infected nodes. Thereafter, infection progresses via a contact network; the probability of a node becoming infected depends on the state of the nodes directly linked to it. The probability *p* of a susceptible node with *k* infectious contacts becoming infected in a small time interval Δ*t* is *p*=1−exp(−*τk*Δ*t*). Here, *τ* is the rate of infection spreading through a single contact between an infectious and a susceptible node.

*Contact-independent identification of infectious nodes I→T*. Disease is detected at an infectious node (e.g. via clinical signs or screening) at rate *α*. This results in the isolation of the node and triggers the tracing of its contacts.

*The removal of triggering nodes T→R*. Triggering nodes are removed at rate *δ*.

*Multi-step contact tracing I→T*. The infectious neighbours of triggering nodes (*T*) can themselves become triggering nodes through contact tracing if they are found to be infected, creating a multi-step contact-tracing chain that tracks the paths of disease transmission. Traced *I* nodes are not directly removed, but instead enter the *T* state at rate *φ*. Unless otherwise stated, we consider multi-step tracing.

*Single-step contact tracing of infectious nodes I→R.* Diagnostic tests are often necessary to determine the status of traced nodes (individuals); these may be imprecise or slow and the isolation and observation of traced individuals may not be a viable option. In such cases, multiple-step contact tracing is less likely. In an alternative single-step contact-tracing model, the infectious neighbours of triggering nodes are traced at rate *φ* per contact. These are then directly removed and do not initiate further tracing. Control is always modelled through either multi-step or single-step tracing.

### 2.2 Individual-based network simulations

Undirected networks with different mixing patterns (assortative and disassortative) are generated using a method proposed by Newman (2003*a*). The mixing pattern here is based on node degree (i.e. the number of links of each node). The level of mixing is given by the correlation coefficient of the ‘excess’ degrees (see below) calculated on all pairs of connected nodes. The generation of networks with different mixing properties is based on a Monte Carlo sampling scheme with repeated link switching at a probability determined by the values of the connectivity matrix *E*=*e*_*ij*_. Here, *e*_*ij*_ is the probability that a randomly chosen link connects a node with *i* connections to a node with *j* connections where the link under consideration is itself not counted. Defining the distribution of the ‘excess’ degree (i.e. degree minus one) of vertices at the end of links as $q_{k}=\sum_{j}e_{jk}$, the level of mixing by vertex degree is given by

(2.1) $$r=\frac{\sum_{jk}jk(e_{jk}-q_{j}q_{k})}{\sigma_{q}^{2}},$$

where $\sigma_{q}^{2}$ is the variance of the distribution *q*_*k*_. For disassortatively mixed networks −1≤*r*<0, for random networks *r*≈0 and for assortatively mixed networks 0<*r*≤1.

Many real networks display a wide distribution of node degrees (Newman *et al.* 2001; Albert & Barabási 2002). To reflect such systems, we consider networks that are generated according to an exponentially truncated power-law degree distribution

(2.2) $$p_{k}=\frac{k^{-\gamma }e^{-k/Κ}}{Li_{\gamma }(e^{-1/Κ})}\;\text{for}\;k\geq 1.$$

The function $Li_{n}(x)$ is the *n*th polylogarithm of *x* and acts as a normalizing constant. The exponential cut-off of the scale-free distribution is determined by *K*. Following Newman (2003*a*), a value of *γ*=2.5 is used for the power-law exponent. The network analysis that illustrates the effect of preferential mixing is performed for *K*=100. For this value of *K*, the average number of connections per node in the networks is 〈*k*〉≈1.7. To explore a range of parameter values that support meaningful epidemics (i.e. where the epidemic is large enough to be of a significant public health concern), 〈*k*〉 must be sufficiently large to allow for a range of new infections in the first generation well above 1. Therefore, the networks are generated by only accepting nodes with *k*≥3 (see Kiss *et al.* 2006*b*).

Moreno *et al.* (2003) proposed a numerical method for epidemic models that can account for connectivity correlations. While this method works without explicitly generating the network, it offers less flexibility when considering disassortatively mixed networks. Here, we use epidemic simulations on networks with *N*=10 000 nodes and consider values of *r*=−0.2, −0.10, −0.05 for disassortatively mixed networks and *r*=0.05, 0.10, 0.2 for assortatively mixed networks. The limits are chosen as the values over which the algorithm appears to be robust (M. E. J. Newman 2006, personal communication); this range of *r* values is sufficient to give marked differences in the epidemic threshold for the transmission rate, number of nodes traced, final epidemic size and to illustrate important trends in contact-tracing efficacy. A further check on the mixing pattern is illustrated in the electronic supplementary material by a plot of the average connectivity of a neighbour as a function of node connectivity for different values of *r*.

All simulated epidemics were seeded with 10 index cases chosen at random, in order to avoid early stochastic extinction. Averages of 10 000 simulations are presented, consisting of 100 epidemic runs on each of 100 different network realizations. Proportions across all plots are relative to the total network size *N*=10 000. The simulation time step used is Δ*t*=0.04. Smaller time steps produced effectively identical results (not shown). The s.e. values of the averages of simulation outputs are at least three to four orders of magnitude smaller than the measurement itself and therefore are not discussed further.

## 3. Results

### 3.1 Giant component size

The giant component (GC) is the largest subset of nodes such that any two nodes from this subset can be connected by a series of links. For undirected networks, the GC size represents the upper limit for the potential size of an epidemic. In figure 2, the structural differences of preferentially mixed networks are demonstrated by the size of the GC as a function of the cut-off parameter *K* for three different values of the assortativity coefficient *r*. As the cut-off parameter increases, the number of links in the network increases and the network becomes denser. A *K*-value exists where the GC size is equal on disassortatively and assortatively mixed networks. Consistent with previous results (Newman 2002, 2003*a*), below this value, GC size is larger on assortatively mixed networks and above it, the GC is larger on disassortatively mixed networks, with GC size generally intermediate on random networks.

The two different regimes are a direct consequence of the mixing pattern. In assortatively mixed networks, nodes of high degree preferentially connect to each other and form a highly connected core group. Therefore, for low link density, a larger GC size is found than for either disassortatively mixed or random networks. Link density within the GC is higher than in the network as a whole. In contrast, in disassortatively mixed networks at low link density, the links are more dispersed, forming many isolated components of small size (figure 3). As link density increases, the probability that a link with one node in the GC will only connect to another node already in the GC also increases; this is a finite size effect that is exacerbated by assortative mixing since high-degree nodes are relatively few. Thus, at higher link densities, assortatively mixed networks have a smaller GC size compared with disassortatively mixed networks, as in the latter, added links are more likely to result in smaller components being absorbed into the GC (figure 2).

### 3.2 Epidemic dynamics

The analysis of the GC growth and component distribution was performed on networks generated using *γ*=2.5 and *k*≥1. For *K*=100, the average number of connections per node is 〈*k*〉≈1.7. For random networks, the percolation transition occurs at 〈*k*〉=1, above which the network will support large epidemics, i.e. epidemics that scale with total population size (e.g. Moore & Newman 2000; Kao *et al.* 2006). Thus, 〈*k*〉≈1.7 supports only a narrow range of epidemiological parameters over which large epidemics can occur. To circumvent this problem, the networks used below were generated using the same parameter values, but only nodes with *k*≥3 were accepted during the network generation process (Kiss *et al.* 2006*b*), resulting in networks with 〈*k*〉≈6.

We define two variables *T*_p_ and *Φ* for ease of reporting and comparing results. With per-contact transmission rate *τ* and detection rate *α*, the transmission probability per link over the period before the detection of the infectious node is given by *T*_p_=*τ*/(*τ*+*α*) (Keeling & Grenfell 2000). Constant 〈*k*〉*T*_p_ provides a constant number of secondary infections caused by the introduction of infection at a node when the remainder of the network is susceptible (Keeling & Grenfell 2000), at the cost of having different epidemic dynamics as 〈*k*〉 varies (Green *et al.* 2006*b*). In a parallel manner, with per-contact-tracing rate *φ* and removal rate of triggering nodes *δ*, the tracing probability per traceable link over the whole tracing triggering period is given by *Φ*=*φ*/(*φ*+*δ*).

In figure 4, in the absence of tracing, the final epidemic size *R*(∞) is plotted against the transmission probability for *r*∈{−0.2, −0.1, −0.05, 0, 0.05, 0.1, 0.2}. In this case, compartments *T* and *R* are equivalent and the current SITR model is equivalent to the well-known SIR model with an effective infectious period of 1/*α*. The relationship between *R*(∞) and *T*_p_ is qualitatively similar to the relationship between GC size and link density in figure 2. The epidemic threshold for assortatively mixed networks occurs at a lower transmission probability than for the other two mixing patterns. However, the final epidemic size approaches its asymptote, total network size, faster for disassortatively mixed networks. The two network types with *r*=−0.2 and 0.2 produce approximately equal epidemic sizes at *τ*≈0.0528 (transmission probability *T*_p_≈0.15), when *R*(∞)≈0.225 on both types.

Epidemics on assortatively mixed networks have a faster initial growth rate and a shorter duration than those on disassortatively mixed networks (figure 5*a*). This is mainly due to the GC containing a ‘core’ group of high-degree nodes that are highly connected. The differences in the initial epidemic growth rate across the different network types are directly related to the basic reproduction number *R*_0_ (Anderson & May 1991). The value of *R*_0_ can be estimated as the lead eigenvalue of the next-generation matrix *C*=(*c*_*ij*_) (Diekmann & Heesterbeek 2000), and, in this case, it can be approximated by the product of the contact matrix and the per-link probability of transmission *c*_*ij*_=*T*_p_*M*_*ij*_. A non-zero entry *M*_*ij*_=1 denotes that an infectious node *j* can transmit the infection to a susceptible node *i*; the magnitude of *c*_*ij*_ is given by the per-link transmission probability *T*_p_. Estimates of *R*_0_ averaged over 100 generated networks of each type are as follows: *R*_0_=12.12*T*_p_ (s.e. 0.11) for disassortatively mixed networks; *R*_0_=15.0*T*_p_ (s.e. 0.11) for random networks; and *R*_0_=17.81*T*_p_ (s.e. 0.14) for assortatively mixed networks. As expected, the threshold for epidemic outbreaks occurs at lower infection probabilities for assortatively mixed networks than for either disassortatively mixed or random networks.

### 3.3 The efficacy of contact tracing

The differences in the initial epidemic growth rate and epidemic duration are likely to have consequences for the efficacy of contact tracing on the different networks. For example, on assortatively mixed networks with fast epidemic turnover, efficient contact tracing has to be comparably fast. The prevalence of traced nodes (dashed line) for assortatively and disassortatively mixed networks is illustrated in figure 5*a*. The average degree of newly infected (Barthélemy *et al.* 2004) and contact-traced nodes is plotted in figure 5*b*. While assortatively mixed networks sustain epidemics with fast turnover and quick initial growth rate, they also allow contact tracing to remove a larger number of highly connected nodes early on in the epidemic. In contrast, on disassortatively mixed networks, disease spread is slower, but contact tracing is also less efficient.

Contact tracing can be viewed as an exploration of the local network structure (Cohen *et al.* 2003), and thus we would expect its efficacy to depend on the mixing patterns of the network. This is investigated by varying *φ*, while keeping *δ* fixed. For comparison purposes, we contrast the cases where *R*(∞) is the same on both assortatively and disassortatively mixed networks, but possibly with differing transmission rates, later concentrating on the unique transmission rate that results in the same *R*(∞) on both networks.

The final epidemic size *R*(∞), the proportion of nodes that become triggering nodes via clinical signs or screening and the proportion of nodes that have been contact traced during the epidemic are plotted in figure 6 as a function of tracing probability *Φ* for disassortatively (*r*=−0.2) and assortatively (*r*=0.2) mixed networks. First, transmission rates are chosen such that *R*(∞)≈0.73 on both networks (*τ*=0.125 and 0.175 on disassortatively and assortatively mixed networks, respectively; figure 6*a*) and, second, the transmission rates are the same (*τ*=0.0528) with *R*(∞)≈0.225 on both networks (figure 6*b*). For the first case, the effect of contact tracing is similar on both networks with comparable *R*(∞); however, we identify two distinct regimes above and below *Φ*=0.61, above which contact tracing becomes more effective on disassortatively mixed networks (figure 6*a*).

In figure 6*a*, if *Φ*≤0.61, *R*(∞) is smaller on assortatively mixed networks. Contact tracing on assortatively mixed networks removes nodes of higher degree (figure 5*b*) than on disassortatively mixed networks. For small *Φ* values, the final epidemic size is still high and the population of highly connected nodes is depleted well before the epidemic ends, as shown by the crossover between the average degree of newly infected nodes on the two network types (figure 5*b*). Over the epidemic, susceptible nodes are on average of lower degree and are more difficult to reach. On disassortatively mixed networks, the depletion of highly connected nodes is less marked and epidemics persist, with disease spread alternating between highly and less well-connected nodes producing an average degree of newly infected nodes which is more even in time (figure 5*b*). In the latter stages of the epidemic, on disassortatively mixed networks, highly connected nodes are not completely depleted and can become infected. Thus, on the assortatively mixed networks, the epidemic ends earlier with a smaller final epidemic size. On assortatively mixed networks, when global depletion of susceptible nodes is important, contact tracing acts to enhance the early depletion of nodes of high degree (figure 5*b*).

At higher tracing probability, the proportion of nodes removed through contact tracing (figure 6*a*) decreases with increasing tracing probability. This is indicative of effective control with a limited proportion of nodes becoming infectious and, hence, fewer targets for tracing. In the regime of more effective control (*Φ*≥0.61), the epidemics die out early on and susceptible depletion becomes less important. This is illustrated in figure 7, showing that the depletion of susceptibles is only found at low *Φ* values. This is also reflected in the very rapid reduction of *R*_g_ (i.e. average reproduction ratio in generation *g* defined as the ratio between the number of nodes infected in consecutive generations) below 1 when *Φ* is high. This is a more general effect corroborated by examining contact-tracing efficacy in a different parameter regime. In figure 6*b*, the scenario where *τ*=0.0528 on both networks is considered. For this value of *τ* and in the absence of tracing, the final epidemic size is the same on both networks (*R*(∞)≈0.225). Here, the final epidemic size is similar to the point at which contact tracing starts to perform better on disassortatively mixed networks in figure 6*a*. In this case, for all *Φ* values, contact tracing always performs better on disassortatively mixed networks. The behaviour in this regime can be explained by interpreting contact tracing as a mechanism acting to reduce the effective transmission probability *T*_p_ past the first generation of infection. Contact tracing achieves this by shortening the average infectious period of traced nodes. In figure 4, lower transmission probabilities correspond to lower values of the transmission rate *τ*. While we do not have exact analytic relationships between the implications of reducing the transmission probability through the different routes, the effect of contact tracing can be approximated by following the trend of the final epidemic size (figure 4) as the transmission probability decreases. The steeper curve of the final epidemic size on disassortatively mixed networks suggests that a small decrease in the transmission probability has a more marked effect on final epidemic size than on assortatively mixed networks. This effect is especially dominant since in the absence of tracing the same *R*(∞) is observed on both networks. This supports the higher contact-tracing efficacy observed on disassortatively mixed networks.

### 3.4 Single-step versus multi-step tracing

Multiple-step contact tracing is not always logistically feasible. It is therefore important to determine the benefit provided by it. For both network types (figure 8), there are considerable differences between the final epidemic sizes for single- and multiple-step contact tracing at high tracing rates, particularly for assortatively mixed networks (figure 8*b*). In multiple-step tracing, a triggering node can generate other triggering nodes, potentially creating a cascade of triggering nodes throughout the infected portion of the network. This leads to tracing a higher number of infectious nodes and reducing the number of links that successfully transmit the disease. For high tracing rates, the epidemics are short-lived, and, on assortatively mixed networks, as a result of the faster initial epidemic growth rate, the extra proportion of untraced infectious nodes in the case of single-step tracing generates a higher number of infections and hence there is a marked difference between single- and multi-step tracing. In figure 8, the faster decrease in the proportion of traced nodes with increasing tracing probability indicates earlier and more effective control in the case of multiple-step contact tracing compared with the single-step case.

## 4. Discussion

Contact tracing performs comparably well on both assortatively and disassortatively mixed networks. This is mainly explained by a balance that is reached between the epidemic time scale (i.e. slow for disassortatively mixed and fast for assortatively mixed; figure 5*a*) and the hierarchy of spread (figure 5*b*) on assortatively mixed networks (and the lack of it on disassortatively mixed networks) on the one hand, and the contact-tracing mechanism. On assortatively mixed networks, the epidemic spread is faster and the disease typically spreads to nodes with high degree. This is counterbalanced by contact tracing that removes infectious nodes with high degree. On disassortatively mixed networks, the epidemic spread is slower; however, owing to the connectivity pattern, contact tracing also alternates between removing poorly and highly connected nodes and therefore is comparably less effective. The higher average degree of traced nodes is a reflection of the average degree of nodes becoming infected earlier on in the epidemic when highly connected nodes are more abundant. This combined with the depletion of highly connected nodes, accentuated by finite size effects, leads to producing a higher average degree of traced nodes when compared with the average degree of infected nodes.

In the case of large epidemics and small values of the contact-tracing rate, contact tracing is more effective on assortatively mixed networks than on disassortative mixed networks, although the difference is small. Here, on the assortatively mixed networks, the early, global depletion of highly connected nodes results in a rapid increase in the proportion of susceptible nodes that are poorly connected nodes, and thus are less likely to become infected. For smaller values of the final epidemic size, the epidemics die out earlier and the depletion of susceptible nodes is less important. The efficacy of contact tracing in this case is determined by the more resilient nature of the assortatively mixed networks to the removal of potentially infectious links through tracing. In the case of single-step versus multi-step contact tracing, the differences are more marked for assortatively mixed networks and small epidemics.

The algorithm used to generate networks with different mixing patterns is robust for mixing values in the range of −0.2 to 0.2. This range covers many of the values measured from networks based on real data (Newman 2002). For more marked differences in the mixing pattern, we expect similar qualitative conclusions with possibly more significant quantitative differences. For the models presented above, differences in contact-tracing efficacy were investigated for various parameter values in addition to those presented. The results agreed qualitatively across the range of parameter values studied. However, further investigation is needed to determine the relative contributions of the different determinants of contact-tracing efficacy. The model presented here does not incorporate time or resource constraints for contact tracing. The implementation of epidemic control strategies often involves qualified personnel and costly or time-consuming diagnostic tests. Although contact tracing performs comparably well on both network types, the faster time course of the epidemic on assortatively mixed networks is more likely to stretch resources in real situations, since it requires a greater and more timely concentration of resources.

As in previous studies investigating the effects of contact clustering (Kiss *et al.* 2005) and degree distribution (Kiss *et al.* 2006*b*) on contact tracing, we show that, unless contact tracing is very good, the mixing patterns have little effect on the course of the epidemic and the number of nodes removed. These results would suggest that it is difficult to exploit network structure to achieve better control via tracing. This may seem somewhat surprising, as previous studies have shown that control strategies such as acquaintance sampling (Cohen *et al.* 2003) that is based on the local exploration of the population contact structure provide an efficient epidemic control strategy, compared with random removal of nodes. However, such studies considered only networks that are randomly mixed and compare random removal with targeted removal (a form of contact tracing). Here, however, we compare targeted removal but where the networks themselves differ. Like the disease itself, contact tracing exploits the local network structure and, for assortatively mixed networks, identifies and removes early the highly connected nodes. This beneficial effect, however, is counterbalanced by the fast initial disease spread to such highly important nodes. Therefore, in the present case, the properties of the network can at the same time enhance disease spread and also increase control efficacy, highlighting the non-trivial interactions between the network structure and the dynamics on networks, showing that added attention is needed when evaluating the efficacy of epidemic control strategies.

## Figures and Tables

**Figure 1:**
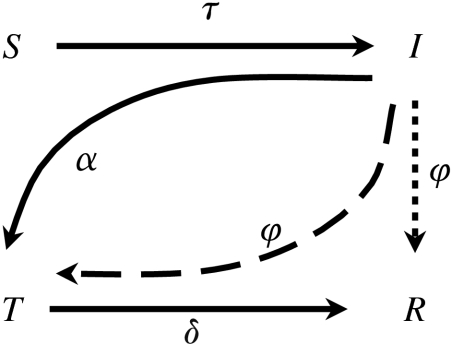
Transitions among the four disease progression states. Contact tracing is represented by either the *I*→*T* transition (multi-step, dashed) or the *I*→*R* transition (single-step, dotted), both with rate *φ*. Tracing occurs through either multi-step or single-step tracing.

**Figure 2:**
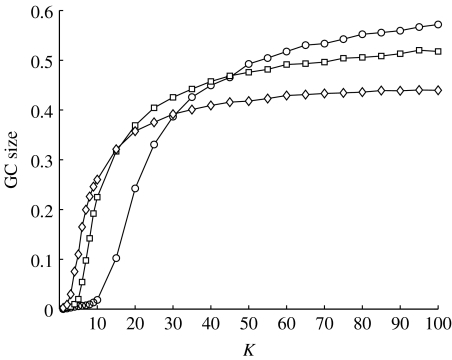
The size of the GC as a function of the cut-off parameter *K* for disassortatively mixed (circles, *r*=−0.15), random (squares, *r*=0) and assortatively mixed (diamonds, *r*=0.15) networks. The average number of connections per node 〈*k*〉 increases with increasing *K*. The parameter values used here are *N*=10 000 and *γ*=2.5.

**Figure 3:**
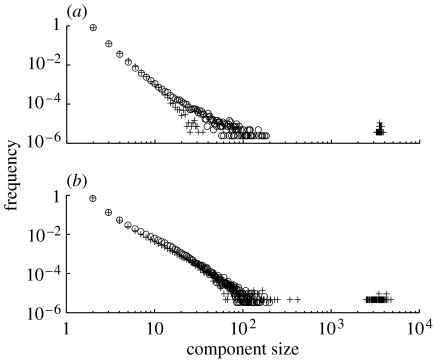
Component size distributions below (circles) and above (pluses) the percolation threshold for (*a*) assortatively mixed and (*b*) disassortatively mixed networks. In disassortatively mixed networks, above the percolation threshold, component sizes are larger than those of assortatively mixed networks. The parameter values used here are *N*=10 000, *γ*=2.5 and *K* is varied to increase link density.

**Figure 4:**
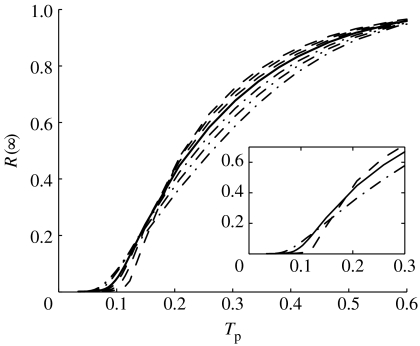
Final epidemic size *R*(∞) in the absence of contact tracing for disassortatively mixed networks (dashed line, *r*≈−0.2, −0.10, −0.05), random networks (solid line, *r*≈0) and assortatively mixed networks (dot-dashed line, *r*≈0.05, 0.10, 0.20) versus probability of transmission *T*_p_=*τ*/(*τ*+*α*) for *N*=10 000, *K*=100, *γ*=2.5, *α*=1/3.5 and *φ*=0.

**Figure 5:**
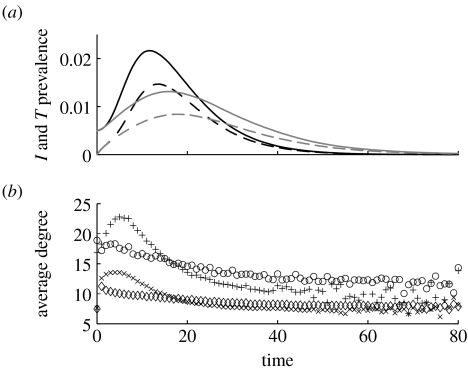
(*a*) Prevalence of infectious (solid lines) and traced (dashed lines) nodes for assortatively mixed (black lines) and disassortatively mixed (grey lines) networks over time. (*b*) The average degree of new infectious (diamonds, crosses) and contact traced *T* (circles, pluses) nodes for disassortatively mixed (diamonds, circles) and assortatively mixed (crosses, pluses) networks over time. Averages are calculated over periods of one time unit. Parameter values are *τ*=0.0528, *α*=1/3.5, *φ*=0.08 and *δ*=1/2.0 with the probability of tracing across a contact of *Φ*=*φ*/(*φ*+*δ*)≌0.138.

**Figure 6:**
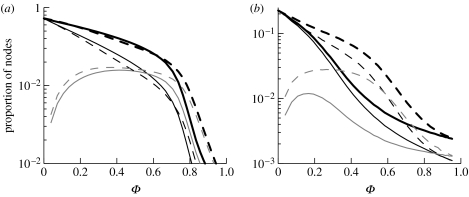
Epidemic properties of disassortatively (solid lines) and assortatively (dashed lines) mixed networks. Final epidemic size (*R*(∞), thick black lines), the proportion of nodes that have become triggering nodes via clinical signs or screening (thin black lines) and the proportion of nodes that have been contact traced over the whole epidemic (grey lines) versus probability of tracing *Φ*=*φ*/(*φ*+*δ*). In (*a*), *τ*=0.125 and 0.175 are used on disassortatively and assortatively mixed networks, respectively. In (*b*), *τ*=0.0528 is used for both network types. Throughout, *α*=1/3.5 and *δ*=1/2.0.

**Figure 7:**
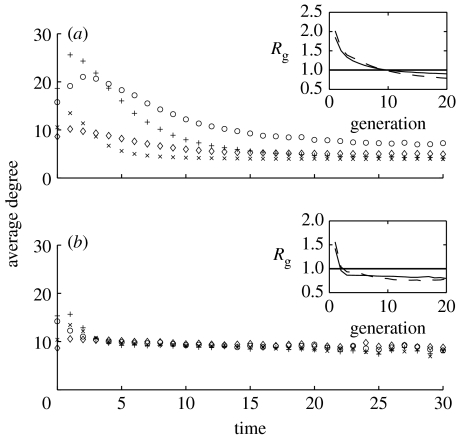
The average degree of new infectious *I* (diamonds, crosses) and contact traced *T* (circles, pluses) nodes for disassortatively (diamonds, circles) and assortatively (crosses, pluses) mixed networks. Averages are calculated over periods of one time unit. Here, *τ*=0.125 and 0.175 are used on disassortatively and assortatively mixed networks, respectively. Throughout, *α*=1/3.5 and *δ*=1/2.0. (*a*) *φ*=0.02 (*Φ*≌0.04) and (*b*) *φ*=2.00 (*Φ*=0.8). The insets show the ratios between the number of infected nodes in consecutive generations (i.e. the average reproduction ratio *R*_g_ in generation *g*) for assortatively (dashed lines) and disassortatively (solid lines) mixed networks.

**Figure 8:**
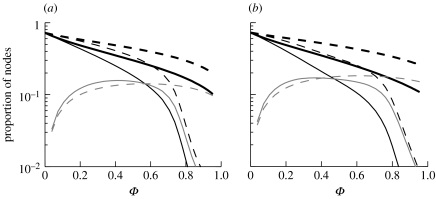
Properties for epidemics with multiple-step tracing (solid lines) and single-step tracing (dashed lines). Final epidemic size (*R*(∞), thick black lines), the proportion of nodes that have become triggering nodes via clinical signs or screening (thin black lines) and the proportion of nodes that have been contact traced during the epidemic (grey lines) versus probability of tracing *Φ*=*φ*/(*φ*+*δ*). Here, *τ*=0.125 and 0.175 are used on (*a*) disassortatively and (*b*) assortatively mixed networks, respectively. Throughout, *α*=1/3.5 and *δ*=1/2.0.

## Supplementary Material

Supplementary figure The effect of network mixing patterns at the level of nodes and links
