# Supplementary material for: The effect of network mixing patterns on epidemic dynamics and the efficacy of disease contact tracing
Source: J R Soc Interface. 2007 Dec 4;5(24):791–9. doi: 10.1098/rsif.2007.1272 (PMC2386895; doi:10.1098/rsif.2007.1272)
Supplement: Supplementary file 1 [file rsif20071272s02.doc]

**Supplementary material for:**

**The effect of network mixing patterns on epidemic dynamics and the efficacy of disease contact tracing**

Istvan Z. Kiss^1,*^, Darren M. Green^2^ and Rowland R. Kao^3^

^1^ Department of Mathematics, University of Sussex, Falmer, Brighton BN1 9RF, UK.

^2^ Institute of Aquaculture, University of Stirling, Stirling, Stirlingshire FK9 4LA, UK.

^3^ Institute of Comparative Medicine, Faculty of Veterinary Medicine, University of Glasgow, Glasgow G61 1QH, UK.

* Corresponding author (i.z.kiss@sussex.ac.uk).

Figure 1: Average connectivity of a neighbour as a function of node connectivity (see Maslov & Sneppen 2002) for disassortatively (top) and assortatively (bottom) mixed networks ($N=10 000$ nodes and $\left\langle k\right\rangle=6$) used in the epidemic simulations.

Fig. 1 illustrates how the measure of preferential mixing$r$, for the whole network, corresponds to the distribution of connections between nodes of various degrees. In disassortatively mixed networks (top panel), the neighbours of poorly connected nodes are more highly connected than neighbours of nodes with many connections. Hence, poorly connected nodes typically connect to highly connected nodes and *vice versa*. In assortatively mixed networks (bottom panel), the opposite is true: nodes that have similar degrees are more likely to be connected. Hence, the neighbours of poorly connected nodes have fewer connections than neighbours of highly connected nodes.

1. Maslov, S. & Sneppen, K. 2002 Specificity and stability in topology of protein networks. *Science* **296**, 910 – 913.
